# Host protein kinases required for SARS-CoV-2 nucleocapsid phosphorylation and viral replication

**DOI:** 10.1126/scisignal.abm0808

**Published:** 2022-10-25

**Authors:** Tomer M. Yaron, Brook E. Heaton, Tyler M. Levy, Jared L. Johnson, Tristan X. Jordan, Benjamin M. Cohen, Alexander Kerelsky, Ting-Yu Lin, Katarina M. Liberatore, Danielle K. Bulaon, Samantha J. Van Nest, Nikos Koundouros, Edward R. Kastenhuber, Marisa N. Mercadante, Kripa Shobana-Ganesh, Long He, Robert E. Schwartz, Shuibing Chen, Harel Weinstein, Olivier Elemento, Elena Piskounova, Benjamin E. Nilsson-Payant, Gina Lee, Joseph D. Trimarco, Kaitlyn N. Burke, Cait E. Hamele, Ryan R. Chaparian, Alfred T. Harding, Aleksandra Tata, Xinyu Zhu, Purushothama Rao Tata, Clare M. Smith, Anthony P. Possemato, Sasha L. Tkachev, Peter V. Hornbeck, Sean A. Beausoleil, Shankara K. Anand, François Aguet, Gad Getz, Andrew D. Davidson, Kate Heesom, Maia Kavanagh-Williamson, David A. Matthews, Benjamin R. tenOever, Lewis C. Cantley, John Blenis, Nicholas S. Heaton

**Affiliations:** 1 Meyer Cancer Center, Weill Cornell Medicine, New York, NY 10021, USA.; 2 Department of Medicine, Weill Cornell Medicine, New York, NY 10021, USA.; 3 Englander Institute for Precision Medicine, Institute for Computational Biomedicine, Weill Cornell Medicine, New York, NY 10021, USA.; 4 Department of Physiology and Biophysics, Weill Cornell Medicine, New York, NY 10065, USA.; 5 Tri-Institutional PhD Program in Computational Biology & Medicine, Weill Cornell Medicine/Memorial Sloan Kettering Cancer Center/The Rockefeller University, New York, NY 10021, USA.; 6 Department of Molecular Genetics and Microbiology, Duke University School of Medicine, Durham, NC 27710, USA.; 7 Cell Signaling Technology, Danvers, MA 01923, USA.; 8 New York University, Grossman School of Medicine, New York, NY 10016, USA.; 9 Weill Cornell Graduate School of Medical Sciences, Cell and Developmental Biology Program, New York, NY 10065, USA.; 10 Division of Gastroenterology and Hepatology, Department of Medicine, Weill Cornell Medicine, New York, NY 10065, USA.; 11 Department of Surgery, Weill Cornell Medicine, New York, NY 10065, USA.; 12 Department of Dermatology, Weill Cornell Medicine, New York, NY 10065, USA.; 13 Department of Microbiology and Molecular Genetics, Chao Family Comprehensive Cancer Center, University of California Irvine School of Medicine, Irvine, CA 92868, USA.; 14 Department of Cell Biology, Duke University School of Medicine, Durham, NC 27710, USA.; 15 Broad Institute of MIT & Harvard, Cambridge, MA 02142, USA.; 16 Department of Pathology, Harvard Medical School, Boston, MA 02115, USA.; 17 Cancer Center and Department of Pathology, Massachusetts General Hospital, Boston, MA 02114, USA.; 18 School of Cellular and Molecular Medicine, University of Bristol, Bristol, BS8 1TD, UK.; 19 Proteomics Facility, University of Bristol, Bristol, BS8 1TD, UK.; 20 Dana-Farber Cancer Institute, Harvard Medical School, Boston, MA 02215, USA.; 21 Department of Cell Biology, Harvard Medical School, Boston, MA 02115, USA.; 22 Department of Pharmacology, Weill Cornell Medicine, New York, NY 10065, USA.; 23 Department of Biochemistry, Weill Cornell Medicine, New York, NY 10065, USA.; 24 Duke Human Vaccine Institute, Duke University School of Medicine Durham, NC 27710, USA.; 25 Duke Cancer Institute, Duke University School of Medicine, Durham, NC 27710, USA.

## Abstract

Multiple coronaviruses have emerged independently in the past 20 years that cause lethal human diseases. Although vaccine development targeting these viruses has been accelerated substantially, there remain patients requiring treatment who cannot be vaccinated or who experience breakthrough infections. Understanding the common host factors necessary for the life cycles of coronaviruses may reveal conserved therapeutic targets. Here, we used the known substrate specificities of mammalian protein kinases to deconvolute the sequence of phosphorylation events mediated by three host protein kinase families (SRPK, GSK-3, and CK1) that coordinately phosphorylated a cluster of serine and threonine residues in the viral N protein, which is required for viral replication. We also showed that loss or inhibition of SRPK1/2, which we propose initiates the N protein phosphorylation cascade, compromised the viral replication cycle. Because these phosphorylation sites are highly conserved across coronaviruses, inhibitors of these protein kinases may not only have therapeutic potential against COVID-19, but also may be broadly useful against multiple coronavirus-mediated diseases.

## INTRODUCTION

In December 2019, a novel human coronavirus, now known as severe acute respiratory syndrome coronavirus-2 (SARS-CoV-2), likely emerged from a zoonotic reservoir and began causing the human disease Coronavirus disease 2019 (COVID-19) (1, 2). Since then, a global pandemic has infected countless numbers of people and caused millions of deaths. Due to the prevalence and severity of this disease, the development of therapeutic interventions is of the highest importance. Much attention has been focused on the development of prophylactic vaccines. Although these vaccines are tremendously effective, their long-term efficacy will likely be limited by the continued adaptation and evolution of the virus.

Antiviral therapeutics address the needs of unvaccinated individuals or those experiencing “breakthrough” infections. Although antiviral development has focused on disrupting the enzymatic activities of viral proteins, targeting host factors that the virus requires to complete its life cycle is also an attractive option. Relative to their hosts, viruses have substantially less coding space in their genomes and therefore utilize the host to enable viral replication. The major advantages of inhibiting a virus indirectly through an essential host factor are two-fold: (i) it has the potential to act broadly because many viruses may utilize the same host protein, and (ii) it is more resistant to evasion by viral mutations compared to direct targeting of the virus, which can rapidly select for resistant viral mutants. Whereas not all host factors are easily targetable, some well-characterized enzymes, such as protein kinases, are of high interest for host-directed antivirals. An essential step in the development of host-directed therapeutics, however, is defining those host factors that are essential for key aspects of the viral replication cycle.

To identify the host kinases critical for SARS-CoV-2, we first broadly profiled phosphorylation sites across viral proteins by phosphoproteomics before focusing on the SARS-CoV-2 nucleocapsid (N) protein. We chose the N protein, and more specifically its SR-rich domain, because of its highly phosphorylated state, conservation across coronaviruses, and the understanding that N protein phosphorylation is important for its functionality (3–16). We then used high-throughput kinase substrate specificity mapping and in vitro phosphorylation assays to define the host kinases that phosphorylate the N protein and the sequential order of phosphorylation at the various sites. To evaluate the necessity of this phosphorylation cascade for the viral life cycle, we used both genetic knockdown and pharmacological targeting of the key protein kinases, serine arginine-rich splicing factor protein kinase 1 and 2 (SRPK1 and SRPK2). We also showed that the FDA-approved kinase inhibitor Alectinib inhibited SRPK1/2 (17), and that this drug can be used to inhibit acute viral infection. Thus, inhibition of the enzymatic activities of SRPK1 and SRPK2, as well as potentially other host kinases, may represent an attractive approach to control SARS-CoV-2 and other coronavirus-mediated infections.

## RESULTS

### The SARS-CoV-2 nucleoprotein is phosphorylated in its SR-rich domain

**Fig. 1.**
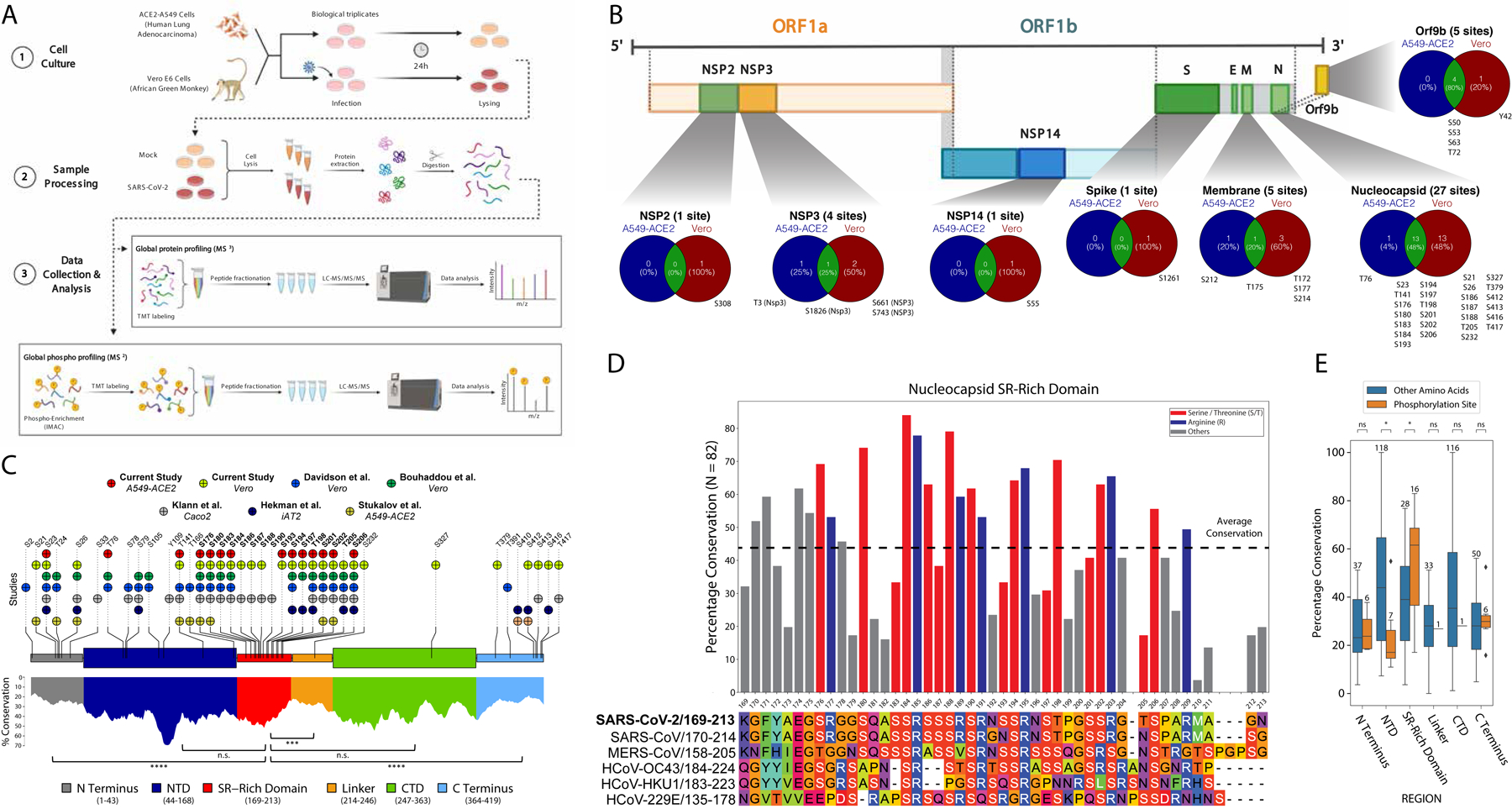
The SARS-CoV-2 N protein is phosphorylated in the SR-rich domain. (**A**) Diagram of the proteomics and phosphoproteomics workflow for cells infected with SARS-CoV-2. (**B**) Phosphorylation sites in SARS-CoV-2 proteins. S, serine; T, threonine, Y, tyrosine. (**C**) Top: Phosphorylation sites in the SARS-CoV-2 N protein identified in seven different phosphoproteomics analyses [two in the current study and five in previously published studies (6–10)]. Bottom: Evolutionary conservation analysis of the different domains of the N protein across 82 different coronaviruses. *P < 0.05, **P < 0.01, ***P < 0.001, and ****P < 0.0001 by nonparametric Wilcoxon-Mann-Whitney U test. (**D**) Percentage identity of amino acids in the SR-rich domain of the N protein across 82 coronaviruses in multiple species (top) and their sequence alignment across six different human coronaviruses (bottom). (**E**) The conservation of the phosphorylation sites in each domain was compared to the conservation of the other amino acids in that domain. The numbers of amino acids compared in each domain are annotated above the boxes. *P < 0.05 by nonparametric Wilcoxon-Mann-Whitney U test.

To identify the phosphorylation sites in the SARS-CoV-2 nucleocapsid (N) protein with high confidence, we infected both human A549 lung epithelial cells expressing the receptor ACE2 (ACE2-A549 cells) and African green monkey (*Chlorocebus sabaeus*) kidney cells (Vero E6 cells) with SARS-CoV-2, because these lines are highly susceptible to infection (18). Infected and control cells were harvested, which was then followed by global proteomic and phosphoproteomic analyses by LC-MS (Fig. 1A). Whereas phosphorylation sites were detected across several different viral proteins (Fig. 1B), the N protein was by far the most phosphorylated. In-depth analysis of the SARS-CoV-2 N protein revealed 14 phosphorylation sites in ACE2-A549 cells, 11 of them specifically in the SR-rich domain (Fig. 1C, Data file S1). In Vero cells, 26 phosphorylation sites were detected in the N protein, 15 of them in the SR-rich domain (Data file S1) in a region in which phosphorylation promotes the gel-to-liquid phase transition of N protein:RNA condensates (16, 19–21). Most of the phosphorylated sites in the SR-rich domain were found in both cell lines in our study, as well as in five previous phosphoproteomics studies (Fig. 1C, top) (6–10). To investigate the evolutionary conservation of the SARS-CoV-2 N protein, we next compared the nucleocapsid proteins from 82 different coronaviruses (Data file S2) and determined the conservation of each amino acid residue. We noticed that the SR-rich domain was significantly more conserved than the linker domain, and as conserved as the two known functional domains of the N protein (Fig. 1C, bottom): the N-terminal domain [NTD, which is involved in RNA binding (22–25)] and the C-terminal domain [(CTD, which is involved in protein oligomerization and RNA binding (26–28)].

Further evolutionary examination of the SR-rich domain showed that the most conserved amino acid residues were serine (S), threonine (T), and arginine (R) (Fig. 1D). Together with previous evidence that the phosphorylation of the SR-rich domain is important for the life cycle of coronaviruses in general (5, 29) and for SARS-CoV (3, 11, 13, 15, 30), we hypothesized that the phosphorylation of the serines and threonines in this basophilic domain was likely to be important for the life cycle of SARS-CoV-2. Finally, comparison of the conservation of the detected phosphorylation sites in each domain to all of the other amino acid residues in that domain showed that only in the SR-rich domain were the phosphorylation sites significantly more conserved relative to those in the rest of the region (Fig. 1E). Together, these data show that not only is the N protein highly phosphorylated, but also that the phosphorylation of the SR-rich domain, in particular, was likely functionally important for the SARS-CoV-2 life cycle.

### Mechanism of nucleoprotein phosphorylation

We next wanted to identify the host kinase(s) that phosphorylated the serines and threonines in the SR-rich domain. Serine/threonine kinases are highly selective with regard to the amino acid sequence that surrounds their serine/threonine phosphoacceptor sites. Most of the human kinases that have been investigated show distinct patterns of selectivities for or against amino acid residues at their phosphorylation sites. This is collectively referred to as their substrate motifs and is useful for identifying biological substrates. To obtain the substrate motif of a kinase, our laboratory and others have developed an unbiased approach using combinatorial peptide substrate libraries (31–34).

**Fig. 2.**
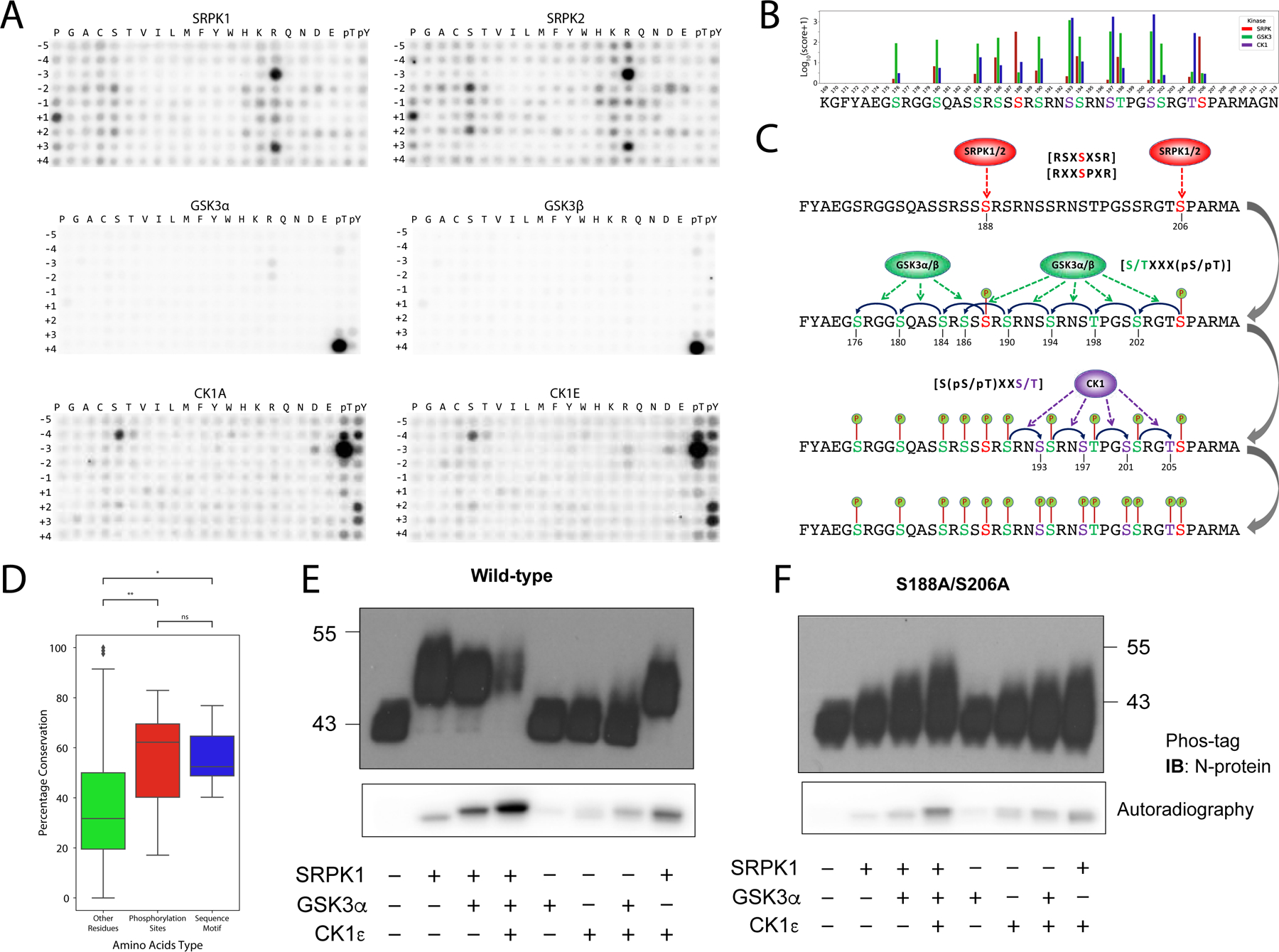
Analysis of the phosphorylation of the SR-rich domain of the N protein by SRPK, GSK-3, and CK1. (**A**) Biochemical substrate specificities of SRPK1/2 (top), GSK-3α/β (middle), and CK1A/ε (bottom). Data are representative of at least two replicate experiments. SRPK isoforms are selective for arginine at the −3 and +3 positions, serine at the −2 and +2 positions, and proline at the +1 position. GSK-3 isoforms are selective for phosphoserine or phosphothreonine at position +4. CK1 isoforms are selective for phosphoserine and phosphothreonine at position −3 and for serine at position −4. (See fig. S1 for the substrate specificities of additional kinases from these families). (**B**) Favorability scores for the different phosphorylation sites in the SR-rich domain according to the SRPK (SRPK1/2/3), GSK-3 (GSK-3α/β), and CK1 (CK1A/D/ε/G1) families. (**C**) Proposed scheme for the multisite phosphorylation of the SR-rich domain of the N protein. Simplified substrate consensus motifs are shown in parentheses, whereas detailed logos are provided in fig. S2. (**D**) Evolutionary conservation comparison of three types of amino acids in the SR-rich domain of the N protein across 82 different coronaviruses from multiple species. Sequence motif: amino acid residues predicted to be essential for the substrate specificity of the priming sites (Ser^188^: Arg^185^/Ser^186^/Ser^190^/Arg^191^; Ser^206^: Arg^203^/Pro^207^/Arg^209^); phosphorylation chains: phosphorylation sites described in the phosphorylation model in Fig. 2C; other amino acids: all other amino acids in the SR-rich domain. *P < 0.05 and **P < 0.01 by nonparametric Wilcoxon-Mann-Whitney U test. (**E**) Top: Western blotting analysis of recombinant N protein on Phos-tag gel after the indicated treatments with the recombinant kinases SRPK1, GSK-3α, and CK1ε. Bottom: SDS-PAGE/autoradiography of recombinant N protein after the indicated treatments with SRPK1, GSK-3α, and CK1ε in the presence of ATP[γ-^32^P]. Data are representative of three experiments. (**F**) Phos-tag gel analysis (top) and autoradiography (bottom) of recombinant N protein with the priming phosphorylation sites mutated (S188A and S206A), which was performed as described fir (E). For comparison, autoradiography of the WT and mutant N proteins was measured together. Data are representative of three experiments.

Previous studies reported that the glycogen synthase kinase-3 (GSK-3) and SRPK families are involved in the phosphorylation of the SR-rich domain of the nucleocapsid protein of SARS-CoV (3, 15). Other reports have suggested that these kinase families are also involved in the phosphorylation of SARS-CoV-2 N protein (9, 35–37). Because of the known preference of SRPKs for serine/arginine-rich proteins (38) and of GSK-3s to phosphorylate serines or threonines four residues C-terminal to the central phosphoacceptor (39), we empirically characterized the biochemical substrate specificities of GSK-3α/β and SRPK1/2/3. Indeed, the SRPKs (SRPK1/2/3) displayed a strong preference for arginine at the −3 and +3 positions, serine at the −2 and +2 positions, and proline at the +1 position (Fig. 2A and figs. S1 and S2). Additionally, and consistent with previous reports (39–42), GSK-3 showed a strong preference for phosphorylating serine or threonine residues that are four residues N-terminal of a previously phosphorylated serine or threonine residue (Fig. 2A and figs. S1 and S2). The phenomenon in which a previously phosphorylated residue promotes the phosphorylation of another proximate residue is called phospho-priming. The SR-rich domain of the N protein contains three potential “chains” of serines/threonines regularly repeating every fourth residue: Ser^206^ to Ser^186^, Thr^205^ to Ser^193^, and Ser^188^ to Ser^176^. We therefore hypothesized that the C-terminal serines/threonines of these chains (the priming sites Ser^206^, T205, and Ser^188^) were phosphorylated first, which would then initiate a phosphorylation cascade by GSK-3, resulting in the full phosphorylation of the chains.

To match each site to its most likely upstream kinase based on the characterized substrate specificity matrices, we computed a favorability score (see Materials and Methods) for the characterized kinases for every phosphorylation site in the three phosphorylation chains described earlier (Fig. 2B and Data file S3). Our algorithm predicted Ser^206^ and Ser^188^ to be phosphorylated by SRPKs (SRPK1/2/3). Once Ser^206^ and Ser^188^ are phosphorylated and thus primed, our algorithm predicted that a GSK-3 family member (GSK-3α/β) would sequentially phosphorylate the chain of serines/threonines every four residues toward the N terminus (Ser^202^-Thr^198^-Ser^194^-Ser^190^-Ser^186^ and Ser^184^-Ser^180^-Ser^176^, respectively).

Finally, because neither the SRPK nor GSK-3 families scored favorably for the third priming site (Thr^205^), we considered additional kinase(s) that might perform this. Assuming that Ser^202^ would be phosphorylated by GSK-3 as discussed earlier, we searched for kinases with a strong preference for phosphoserine or phosphothreonine at position −3 as the most likely kinases to phosphorylate Thr^205^. The CK1 family is a second group of phosphopriming-dependent protein kinases that strongly favors phosphoserine or phosphothreonine at position −3 and partially selects for an unmodified serine at the −4 position (Fig. 2A and fig. S1). In parallel, our algorithm predicted Thr^205^ to be a likely phosphorylation site for CK1, when primed by phosphorylation at Ser^202^ (Fig. 2B and Data file S3). Subsequent phosphorylation at Ser^201^, Ser^197^, and Ser^193^ could be performed by CK1 or GSK-3. We summarized our proposed model for the cluster of phosphorylation sites in the SR-rich domain of the SARS-CoV-2 N protein (Fig. 2C). The amino acid residues that are dominant in the substrate motif of SRPK (Ser^188^: Arg^185^/Ser^186^/Ser^190^/Arg^191^; Ser^206^: Agr^203^/Pro^207^/Arg^209^) are as conserved as the phosphorylated residues across the family of coronaviruses, suggesting that these residues indeed play an important role in directing the appropriate protein kinases to phosphorylate this region of the N protein and that this phosphorylation cascade is critical for the life cycle of this broad family of viruses (Fig. 2D and fig. S3).

To experimentally test our sequential phosphorylation model, we purified recombinant SARS-CoV-2 N protein, incubated it with recombinant SRPK1, GSK-3α, and CK1ε, and performed in vitro phosphorylation assays (Fig. 2E). Phos-tag gel analysis of the reactions showed an upward shift of the N protein band after treatment with SRPK1, indicating stoichiometric phosphorylation at one or more sites of the N protein. Adding GSK-3 or CK1 without previous treatment with SRPK1 had only modest effects on the phos-tag shift. To determine the amount of phosphate incorporation into the N protein, radioactively labeled ATP was included in the phosphorylation reactions, and autoradiography of the N protein was measured by SDS-PAGE. Treatment with SRPK, GSK-3, and CK1 increased the phosphorylation of N protein to a greater extent than the sum of the individual kinase reactions, consistent with our model in which SRPK primes the SR-rich region for phosphorylation by GSK-3 and CK1 (Fig. 2E). Adding all three kinases caused a substantial upward shift in the Phos-tag gel and resulted in reduced detection of protein, suggesting that the highly phosphorylated protein did not efficiently enter the gel (Fig. 2E). The phospho-null double mutant (S188A, S206A) abolished the phos-tag shift caused by SRPK and showed reduced radioactive incorporation by SRPK, GSK-3, and CK1 (Fig. 2F). This is consistent with our model that phosphorylations of the N protein at Ser^188^ and Ser^206^ by SRPK are the critical priming events for extensive phosphorylation of the SR-rich domain.

### Inhibition of SRPK1 results in a decrease in viral propagation

**Fig. 3.**
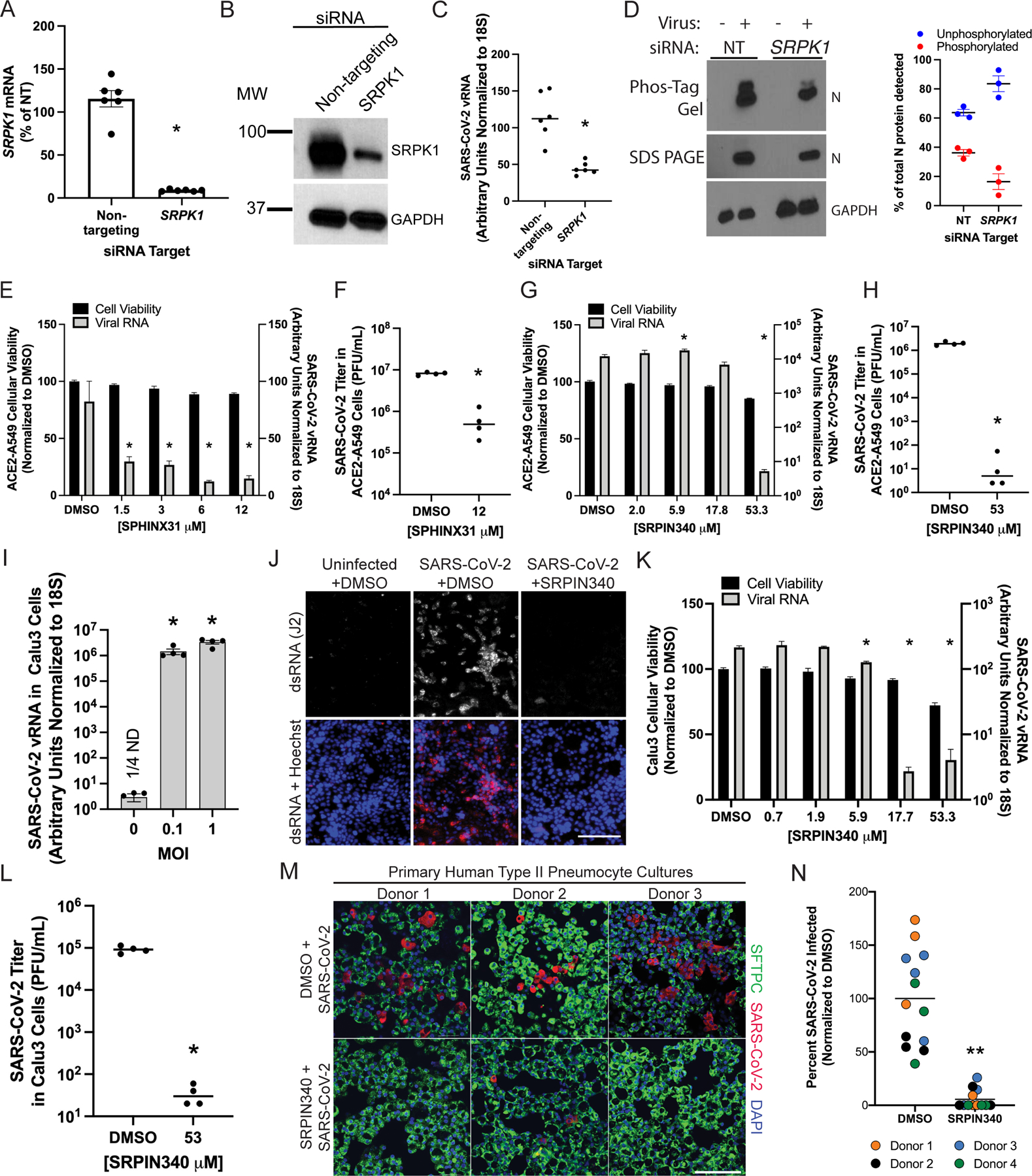
SRPK1/2 inhibitors suppress SARS-CoV-2 infection. (**A**) qRT-PCR analysis of the relative abundance of *SRPK1* mRNA in cells treated with nontargeting (NT) or *SRPK1*-specific siRNA ACE2-A549 cells. Data are means + SEM of six independent biological replicates. (**B**) Western blotting analysis of cells treated with nontargeting or *SRPK1*-specific siRNA. Blots are representative of three experiments. (**C**) SARS-CoV-2 viral RNA was measured by qRT-PCR assay 24 hours after the infection of ACE2-A549 cells that were treated with nontargeting or *SRPK1*-specific siRNA. Data are from six independent biological replicates. (**D**) Left: Western blotting analysis of cells treated with nontargeting or *SRPK1*-specific siRNA and then infected with SARS-CoV-2. Lysates were resolved on Phos-Tag gels or SDS PAGE gels. Blots are representative of three experiments. Right: Relative percentages of phosphorylated and unphosphorylated N protein quantified across replicate Western blots with ImageJ software. Data are from three experiments. (**E**) Cellular viability (left axis) was measured after ACE2-A549 cells were treated with the indicated concentrations of SPHINX31. Data are from four independent biological replicates. SARS-CoV-2 viral RNA abundance (right axis) was measured by qRT-PCR analysis 24 hours after infection of cells. Data are from four independent biological replicates. (**F**) Infectious viral titers were measured by plaque assay of the supernatants of cells that were treated with SPHINX31 and infected for 48 hours. Data are from four independent biological replicates. (**G**) Cellular viability (left axis) was measured after ACE2-A549 cells were treated with the indicated concentrations of SRPIN340 before infection. Data are from six independent replicates. SARS-CoV-2 viral RNA (right axis) was measured by qRT-PCR analysis 24 hours after infection. Data are from four independent biological replicates. (**H**) Infectious viral titers were measured by plaque assay of the supernatants of cells that were treated with SRPIN340 and infected for 48 hours. Data are from four independent biological replicates. (**I**) Calu-3 cells were infected with SARS-CoV-2 at the indicated MOI and SARS-CoV-2 viral RNA abundance was quantified by qRT-PCR after 24 hours of infection. Data are from four independent biological replicates; ND, not detected. (**J**) Calu-3 cells treated with DMSO as a control or SRPIN340 were infected with SARS-CoV-2 at an MOI of 2.5. Twenty-four hours after infection, the cells were fixed and stained for DNA with Hoechst and dsRNA with the J2 antibody. Scale bar, 150 μm. Images are representative of at least two images and two independent experiments. (**K**) Cellular viability (left axis) was measured after Calu-3 cells were treated with the indicated concentrations of SRPIN340, and SARS-CoV-2 viral RNA abundance (right axis) was measured by qRT-PCR analysis 24 hours after infection. Data are from four independent biological replicates. (**L**) Infectious viral titers were measured by plaque assay of the supernatants of Calu-3 cells treated with SRPIN340 and infected for 48 hours. Data are from four independent biological replicates. (**M**) Representative primary human type II pneumocyte culture images from samples that were pre-treated with SRPIN340 for 12 hours before they were infected with SARS-CoV-2. Twenty-four hours later, the cells were fixed and stained for SARS-CoV-2, DNA with DAPI, and surfactant protein C (SFTPC). Scale bar, 100 μm. (**N**) Quantification of the percentages of cells from the experiment described in (M) that were infected with SARS-CoV-2. At least three images each from the of four independent human donors are shown. For all panels, error bars represent SEM and statistical significance was calculated by nonparametric Wilcoxon-Mann-Whitney U test: *P < 0.05, **P < 0.001.

To determine how the loss of SRPK proteins would affect viral replication, we first targeted *SRPK1* in our ACE2-A549 cells (fig. S4, A to D) with RNAi. Both SPRK1 mRNA and protein were significantly reduced in abundance after treatment with RNAi (Fig. 3, A and B), and viral RNA amounts were correspondingly reduced (Fig. 3C). To test our prediction that in the absence of SRPK1, N protein phosphorylation would be decreased, we infected ACE2-A549 cells that were previously treated with a nontargeting siRNA or an SRPK1-targeting siRNA pool. We then ran a Phos-tag gel with lysates from those cells and used an anti-N antibody to detect the SARS-CoV-2 N protein (Fig. 3D, left). When we loaded equal amounts of SARS-CoV2 N protein, which was confirmed by Western blotting analysis, we observed two bands for the N protein: an upper band corresponding to the phosphorylated N protein and a lower band corresponding to the unphosphorylated N protein. When SRPK1 was knocked down with siRNA, there was a decrease in the protein band that corresponded to the phosphorylated N protein (Fig. 3D, right).

As an orthogonal approach to test the requirement of SRPK1 during SARS-CoV-2 infection, we used the known SRPK inhibitor SPHINX31 to block SRPK1/2 activity (43). After treatment of ACE2-A549 cells with SPHINX31, which inhibits both SRPK1 and SRPK2, we observed inhibition of SARS-CoV2 replication (Fig. 3, E and F). Treatment with a second SRPK1/2 inhibitor, SRPIN340 (44), also led to decreased viral RNA and infectious viral titer in a dose-dependent manner at concentrations that were well tolerated by the cells (Fig. 3, G and H). Because ACE2-A549 cell lines are an artificial SARS-CoV-2 infection system, we repeated the SRPK1/2 inhibitor experiments with naturally infectible Calu-3 cells (Fig. 3I) and primary human pneumocytes. Similar to the ACE2-A549 experiments, SARS-CoV-2 infection and replication were significantly inhibited as measured by immunofluorescence of the viral replication intermediate, viral RNA, and cell-free infectious viral particles (Fig. 3, J to N). Although we cannot rule out potential effects of these inhibitors on non-N related aspects of the SARS-CoV-2 life cycle, together, the data are consistent with our model for N protein phosphorylation.

### An FDA-approved SRPK1/2 inhibitor results in decreased viral propagation and N protein phosphorylation

**Fig. 4.**
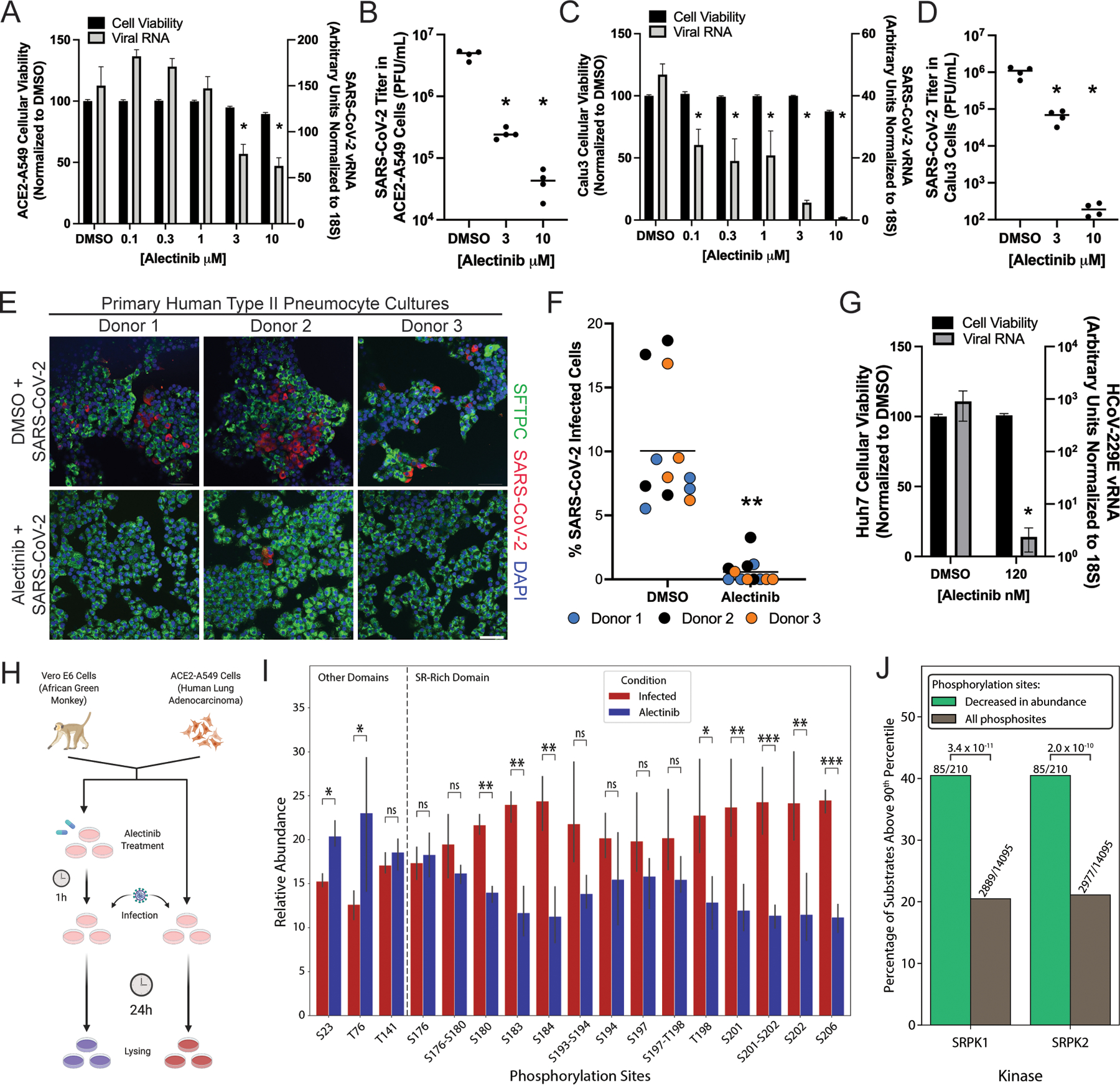
The FDA-approved kinase inhibitor Alectinib inhibits SARS-CoV-2 infection and reduces the extent of phosphorylation of the N protein. (**A**) Cell viability (left axis) was measured after ACE2-A549 cells were treated with the indicated concentrations of Alectinib before being infected. SARS-CoV-2 viral RNA abundance (right axis) was measured by qRT-PCR assay 24 hours after infection. Data are from four independent biological replicates. (**B**) Infectious viral titers were measured by plaque assay of the supernatants of ACE2-A549 cells that were treated with Alectinib and infected for 48 hours. Data are from four independent biological replicates. (**C**) Cell viability (left axis) was measured after Calu-3 cells were treated with the indicated concentrations of Alectinib. SARS-CoV-2 viral RNA abundance (right axis) was measured by qRT-PCR assay 24 hours after infection. Data are from four independent biological replicates. (**D**) Infectious viral titers were measured by plaque assay of the supernatants of Calu-3 cells that were treated with Alectinib and infected for 48 hours. Data are from four independent biological replicates. (**E**) Representative images from primary human type II pneumocyte cultures treated with Alectinib for 12 hours before infection with SARS-CoV-2. Twenty-four hours later, the cells were fixed and stained for SARS-CoV-2 S protein, DNA (with DAPI), and SFTPC. Scale bar, 50 μm. (**F**) Quantification of the percentages of cells from the experiment described in (E) that were infected with SARS-CoV-2. Four independent images for each of the three different human donors are shown. (**G**) Cell viability (left axis) was measured after HuH7 cells were treated with Alectinib before infection. The relative abundance of 229E viral RNA was measured by qRT-PCR assay 24 hours after infection. Data are from four independent biological replicates. For all panels, statistical significance was calculated by nonparametric Wilcoxon-Mann-Whitney U test, unless otherwise stated, and measurements were taken from distinct samples; *P < 0.05, **P < 0.001. (**H**) Scheme for Alectinib treatment and infection for proteomics and phosphoproteomics analysis. Experiments were performed three times. (**I**) Relative abundance (normalized by total protein abundance) of the different phosphorylated sites in the N protein from ACE2-A549 cells treated with or without Alectinib before infection with SARS-CoV-2. Adjusted *P* values (FDR) were computed by moderated *t* test and adjusted by Benjamini-Hochberg correction. *FDR < 0.1, **FDR < 0.05, ***FDR < 0.01. (**J**) Comparison of the frequency of sites that scored high for SRPK1 and SRPK2 (>90^th^ percentile) among phosphorylation sites that were decreased in abundance upon Alectinib treatment and their frequency among all the detected phosphorylation sites in ACE2-A549 cells. Denoted *P* values were computed by Fisher’s exact test.

In the interest of eventual human translatability, we wanted to evaluate an FDA-approved SRPK1/2 inhibitor for its effects on viral replication. Although there are no approved drugs that specifically inhibit SRPK1/2, the anaplastic lymphoma kinase (ALK) inhibitor Alectinib, which is used clinically to treat nonsmall-cell lung cancer, also leads to inhibition of SRPK1/2 (17). Treatment of ACE2-A549 cells, Calu-3 cells, and primary human pneumocytes with Alectinib led to a significant reduction in both viral RNA and infectious titer in a dose-dependent manner (Fig. 4, A to F). Because the SR-rich domains of N proteins from diverse human coronaviruses are highly conserved (45, 46), we also infected Alectinib-treated cells with the alphacoronavirus HCoV-229E (which is distantly related to the beta-coronavirus SARS-CoV-2) and observed inhibition of HCoV-229E by more than 1,000-fold (Fig. 4G). These data suggest that the requirement for SRPK1/2 activity is not restricted to SARS-CoV-2 or even betacoronaviruses. Thus, an FDA-approved kinase inhibitor might be re-purposed as a broadly acting anti-coronavirus therapeutic.

Finally, we wanted to ensure that the inhibitory properties of Alectinib were associated with a reduction in N protein phosphorylation. We therefore collected Alectinib-pretreated, infected ACE2-A549 and Vero E6 cells, performed proteomic and phosphoproteomic analysis, and compared the data to those discussed earlier (Fig. 4H). As expected from the viral inhibition experiments, Alectinib reduced the amounts of viral proteins (fig. S5 and Data file S4). Furthermore, the phosphoproteomics data revealed that most of the SR-rich domain phosphorylation events were reduced upon Alectinib treatment, whereas the extent of phosphorylation of sites outside the SR-rich domain did not decrease (Fig. 4I, fig. S6A, Data file S5). We then scored all of the detected (host and viral) phosphorylation sites by SRPK substrate specificity matrices and examined the sites that showed decreased phosphorylation upon Alectinib treatment. The proportion of sites that scored highly for the SRPK family (scoring above the 90^th^ percentile) among the phosphorylation sites that were reduced in abundance was significantly greater than their proportion among all of the measured phosphorylation sites (Fig. 4J and fig. S6B), providing further evidence that Alectinib inhibited members of the SRPK family and that phosphorylation of the viral N protein was affected as anticipated.

## DISCUSSION

Our study of N protein phosphorylation led to the identification of SRPK1 and SRPK2 as kinases that are critical for the replication of coronaviruses as divergent as 229E and SARS-CoV-2. Furthermore, the phosphorylation sites in the SR-rich domain of the N protein and their surrounding sequences are also conserved among bat coronaviruses (fig. S3), suggesting that these kinases may also be targetable for pre-pandemic coronaviruses. Whereas SRPK1 and SRPK2 are found in most human tissues and have been implicated in a number of basal processes, including the regulation of transcript splicing, lipid metabolism, and cellular stress responses (47–59), their activity is also important for the replication of viruses, such as hepatitis B virus, human papillomavirus, hepatitis C virus, SARS-CoV, Ebola virus, human cytomegalovirus, and herpes simplex virus-1 (15, 60–67). Although the mechanisms underlying how SRPK1/2 contribute to the replication of these viruses differ, it appears that many viruses have evolved to take advantage of these host protein kinases.

Posttranslational modification of viral proteins is well understood to be important for the functionality of viral proteins, with phosphorylation chief among them (5, 68). At least two reports have implicated different kinases, including growth factor receptor (GFR)–activated kinases, casein kinase II (CK2), cyclin-dependent kinases (CDKs), and protein kinase C (PKC), as generally important for SARS-CoV-2 replication, but not necessarily directly linked to N protein phosphorylation (6–9, 69, 70). Previous work with the related betacoronavirus SARS-CoV showed that, at least in vitro, CDK, GSK, mitogen-activated protein kinase (MAPK), SRPK1, and CK2 can phosphorylate the SARS-CoV N protein (15, 71). Here, whereas we characterized several of the kinases that are important for phosphorylation of the SARS-CoV-2 N protein SR-rich domain, note that other kinases might also play key roles in the phosphorylation of other N protein domains and viral proteins. Understanding the relative contributions of other potential kinases (and their potential redundancy) to the phosphorylation of different viral proteins and indeed, different domains within the same protein, remains an important area of future study.

Future work will also be needed to establish the functional role of N protein phosphorylation in the SARS-CoV-2 replication cycle. Studies have reported that SARS-CoV-2 N protein phosphorylation can affect its protein-protein and protein-RNA interactions, as well as its gel-liquid phase transition activities (16, 20), functions that may also affect NF-κB activation (72). Phosphorylation by SRPK1 affects the ability of the SARS-CoV N protein to multimerize, although effects on viral growth were not reported (15). Additionally, the replication of both SARS-CoV and mouse hepatitis virus (MHV) are suppressed after treatment with GSK-3 inhibitors, presumably at least partially by affecting N protein phosphorylation (3, 14). At least for SARS-CoV-2, it appears that SRPK1/2 are central to the regulation of viral replication despite the fact that the full range of effects resulting from incomplete N phosphorylation remains unclear.

More broadly, whereas our study has focused on the phosphorylation of the SR-rich domain of the viral N protein, we cannot rule out that viral inhibition may be at least partially due to the altered phosphorylation of host proteins or other viral proteins. Indeed, our proteomic analysis revealed that after Alectinib treatment, the abundances of some antiviral products of interferon-stimulated genes increased. This could mean that SRPKs (or other kinases inhibited by Alectinib) are important for the normal expression of antiviral genes or that inhibition of N phosphorylation compromises viral immune suppression activities. Although our experiments do not discriminate between these possibilities, there are independent reports of the N protein suppressing host protein translation and altering the antiviral immune response (15, 73). Additionally, whereas we found that SRPK1/2 inhibitors were effective in both immortalized and primary human cells, future studies will be required to determine whether targeting SRPK1/2 in vivo has a similar magnitude of effect. Although not definitive, a favorable outcome of COVID-19 was reported in two cases of patients with non-small cell lung cancer (NSCLC) administered with Alectinib (74, 75).

Even as efficacious vaccines control the spread of SARS-CoV-2, the development of antiviral therapeutics remains a relevant goal to protect those who experience breakthrough infections or, for various reasons, are not immunized. Outside of COVID-19 specifically, host-directed therapeutics may also be important in the event of future outbreaks provided that emerging viruses use the same host factors. This study has characterized the activities of several kinases that may be broadly required for many coronaviruses, and, at least for one, an FDA-approved inhibitor already exists. Our data showed that not only was viral replication compromised after inhibition of kinase activity, but also there was a generally heightened antiviral response. Thus, inhibition of host kinases could both suppress active viral replication and enhance the natural ability of the host to block infection. Continued development of inhibitors of SRPK1/2 and other kinases (potentially in combination) may lead to a class of broadly acting, host-directed antiviral therapeutics that could help combat coronavirus-mediated disease.

## MATERIALS AND METHODS

### Cell culture

At Duke University, all cells were obtained from ATCC and grown at 37°C in 5% CO_2_. 293T cells (ATCC CRL-11268), A549 cells (ATCC CCL-185), and Huh7 cells (a kind gift of Dr. Emily Derbyshire – Duke University) cells were grown in DMEM with 10% FBS, Glutamax, and penicillin/streptomycin. To express ACE2 in A549 cells, both unmodified A549 cells and A549 cells harboring Cas9 were transduced with a VSV-G pseudotyped lentivirus expressing hACE2, and both of the resulting lines were used for experimentation. Vero E6 cells were grown in MEM supplemented with Penicillin/Streptomycin, 10% FBS, 1 mM Pyruvate, and 1X MEM NEAA. Calu-3 cells were grown in EMEM with 10% FBS and Penicillin/Streptomycin. At ISMMS, Vero E6 cells were obtained from ATCC (CRL-1586). A549 cells stably expressing the SARS-CoV-2 receptor ACE2 were previously described (76). All cells were maintained in DMEM supplemented with 10% FBS and Penicillin/Streptomycin at 37°C in 5% CO_2_.

### SARS-CoV-2 infections and titering

A stock of BEI isolate SARS-CoV-2 USA-WA1/2020 (a kind gift of Greg Sempowski, Duke University) was grown on VeroE6 cells in viral growth medium (MEM supplemented with 1% Pen/Strep, 2% FBS, 1 mM Sodium Pyruvate, and 1x MEM NEAA) for the experiments in Figs 3 and 4 and fig. S4. For viral stock production, virus was incubated with the cells for 1 hour at 37℃. After infection, the total volume of medium was brought to 30 ml. Virus was harvested after 72 hours of infection. To determine the viral titer of the stock and after drug treatment, a monolayer of VeroE6 cells was infected with serially diluted virus for 1 hour. The virus was then removed and an agar overlay was added to each well (MEM, Penicillin/Streptomycin, 2% FBS, 1 mM Pyruvate, 1x MEM NEAA, 0.3% Sodium Bicarbonate, Glutamax, 0.7% oxoid agar). Plaque assays were incubated for 72 hours and then stained with either 0.1% Crystal Violet in 10% neutral-buffered formalin (NBF) or 0.05% Neutral Red in phosphate-buffered saline (PBS). As has been reported for other SARS-CoV-2 stocks grown on Vero cells, our viral stocks were a mixed population harboring both full-length and furin cleavage site deletion spike (S) genes. All viral infections took place in cell-specific medium with only 2% FBS.

### HCoV-229E infections and titering

A stock of isolate HCoV-229E VR-740 (ATCC) was grown on Huh7 cells in complete medium (DMEM supplemented with 10% FBS, 1% Pen/Strep, Glutamax). Infection of cells was performed for 1 hour at 37°C. After infection, the total volume of the medium was brought to 20 ml. Virus was harvested after 36 hours of infection. The viral titer of the stock was determined by plaque assays on Huh7 cells. A confluent monolayer of cells was infected with serial dilutions of virus in complete medium for 1 hour at 37°C. Virus was removed, and an agar overlay was added to each well (DMEM, 10% FBS, 1% Pen/Strep, Glutamax, 0.5% oxoid agar). Plaque assays were incubated for 72 hours and then stained with 0.1% Crystal Violet in PBS. Viral infection assays took place in complete medium (DMEM, 10% FBS, 1% Pen/Strep, Glutamax).

### SARS-CoV-2 infection for phosphoproteomics analysis

SARS-CoV-2 isolate USA-WA1/2020 (NR-52281) was deposited by the Center for Disease Control and Prevention and obtained through BEI Resources, NIAID, NIH. SARS-CoV-2 was propagated in Vero E6 cells (ATCC, CRL-1586) in DMEM supplemented with 2% FBS, 4.5 g/L D-glucose, 4 mM L-glutamine, 10 mM Non-Essential Amino Acids, 1 mM Sodium Pyruvate, and 10 mM HEPES as previously described (77). Viral stocks were filtered and cleared from cytokines and other contaminating host factors by centrifugation through Amicon Ultra-15 100K Centrifugal Filter Units before use. For phosphoproteomic analysis, 6×10^7^ Vero E6 or 6×10^7^ ACE2-A549 cells were treated with 5 µM Alectinib or DMSO in DMEM supplemented with 2% FBS, 4.5 g/L D-glucose, 4 mM L-glutamine, 10 mM Non-Essential Amino Acids, 1 mM Sodium Pyruvate and 10 mM HEPES for 1 hour at 37℃ before infection. Cells were subsequently infected with SARS-CoV-2 at an MOI of 0.5 for 24 hours at 37℃. After two washes with PBS and removal of all cell culture medium, cell monolayers were lysed in lysis buffer containing 9 M urea, 20 mM HEPES (pH 8.0), with 2X phosphatase inhibitors (Cell Signaling Technology #5870).

### Lysis, digestion, and preparation of samples for mass spectrometry analysis

Cultured cells were rinsed with PBS, and scraped into lysis buffer containing 9 M urea, 20 mM HEPES (pH 8.0), with 2X phosphatase inhibitors (Cell Signaling Technology #5870). Samples were probe-tip sonicated and subsequently reduced with 5 mM DTT for 50 min at 55°C, alkylated for 30 min with 10 mM iodoacetamide, and quenched with 5 mM DTT. Samples were diluted to 2M urea with digestion dilution buffer [20 mM HEPES (pH 8.5) containing 1mM CaCl_2_) and digested at 37^°^C with 20 µg of Lysyl Endopeptidase (Wako-Chem) overnight. Samples were then diluted to 1 M urea and digested for 5 hours with 20 µg of trypsin (Pierce). After digestion, peptides were acidified with trifluoroacetic acid (TFA), centrifuged at 500 *g* for 20 min, and purified over SepPak C18 columns. After elution, peptides were quantified with a MicroBCA assay (Thermo Fisher Scientific).

### Total protein sample preparation

Fifty micrograms of peptides from each sample were labeled with isobaric tandem-mass-tag (TMT) 11 plex reagents (Thermo Fisher Scientific) in 20 mM Hepes (pH 8.5) with 30% acetonitrile (v/v) and 250 μg of TMT reagent. The reaction was quenched for 15 min by adding hydroxylamine to a final concentration of 0.3% (v/v). Samples were combined, dried, purified over SepPak C18 columns, and dried again. Samples were then resuspended in 40 μl of basic reverse phase (bRP) buffer A [10 mM NH_4_HCO_2_ (pH 10), 5% ACN) and separated on a Zorbax Extended C18 column (2.1 × 150 mm, 3.5 µm, no. 763750-902, Agilent) with a gradient of 10 to 40% bRP buffer B [10 mM NH_4_HCO_2_ (pH 10), 90% ACN). Ninety-six fractions were collected and combined into 24 fractions for analysis. Each fraction was dried and desalted over a C18 stop-and-go extraction tip (STAGE-Tip) before analysis by mass spectrometry.

### IMAC phosphopeptide sample preparation

High-Select Fe-NTA Phosphopeptide Enrichment Kits (Thermo) were used to enrich phosphopeptides from 1 mg of peptides for each sample. After elution from the IMAC column, enriched samples were dried, labeled with TMT, and bRP-fractionated as described earlier but with a gradient of 5 to 40% bRP buffer B. The resulting 24 fractions were desalted over a C18 STAGE-Tip.

### LC-MS analysis of total protein fractions

Samples were analyzed on an Orbitrap Fusion Lumos mass spectrometer (Thermo Fisher Scientific) coupled with a Proxeon EASY-nLC 1200 liquid chromatography (LC) pump (Thermo Fisher Scientific). Peptides were separated on a 100-μm inner diameter microcapillary column packed with ~40 cm of Accucore150 resin (2.6 μm, 150 Å, ThermoFisher Scientific). For each analysis, approximately 1 μg was loaded onto the column. Peptides were separated with a 2.5-hour gradient of 6 to 30% acetonitrile in 0.125% formic acid with a flow rate of 550 nl/min. Each analysis used an SPS-MS3-based TMT method (78, 79), which reduces ion interference compared to MS2 quantification (80). The scan sequence began with an MS1 spectrum [Orbitrap analysis, resolution 60,000; 350 to 1400 m/z, automatic gain control (AGC) target 4.0 × 10^5^, maximum injection time: 50 ms]. Precursors for MS2/MS3 analysis were selected with a Top10 method. MS2 analysis consisted of collision-induced dissociation [quadrupole ion trap; AGC 2.0 × 10^4^; normalized collision energy (NCE) 35; maximum injection time: 120 ms]. After the acquisition of each MS2 spectrum, an MS3 spectrum was collected through a method in which multiple MS2 fragment ions were captured in the MS3 precursor population using isolation waveforms with multiple frequency notches (79). MS3 precursors were fragmented by HCD and analyzed with the Orbitrap (NCE 65, AGC 3.5 × 10^5^, maximum injection time: 150 ms; isolation window: 1.2 Th; resolution was 50,000 at 200 Th).

### LC-MS analysis of phosphopeptide-enriched fractions

For phosphopeptide analysis, the same MS and HPLC instruments were used as described earlier. For each analysis, approximately 500 ng of enriched peptides were loaded on the column and run over a 120-minute gradient of 2 to 32% acetonitrile in 0.125% formic acid with a flow rate of 400 nl/min. MS1 spectra were collected in the Orbitrap at a resolution of 60,000 with a scan range of 300 to 1500 m/z using an AGC target of 4.0 × 10^5^ with a maximum inject time of 25 ms. Peptides for MS2 analysis were isolated using the quadrupole with an isolation window of 0.5 m/z. MS2 spectra were generated using Higher-energy collision dissociation (HCD) with a collision energy of 40%. Fragments were collected in the Orbitrap at a resolution of 50,000 with a first mass of 110m/z, an AGC target of 1 × 10^5^, and a maximum injection time of 200 ms.

### Total protein data processing and analysis

Mass spectra were processed with a Comet-based software pipeline (81, 82). The resulting data were searched with a fully tryptic database containing both human Swissprot consensus entries and isoforms downloaded in February 2020, and SARS-CoV-2 pre-release entries from Uniprot (June 2020), enabling a static modification of lysine and N-termini with TMT (229.1629 Da) and carbamidomethylation (57.0215 Da) of cysteine, together with variable oxidation (15.9949 Da) of methionine. Vero cell data were searched with a fully tryptic database containing *Chlorocebus sabaeus* entries from Uniprot (June 2020) and SARS-CoV-2 pre-release entries from Uniprot (June 2020). Searches were performed with a 20-ppm precursor ion tolerance. The product ion tolerance was set to 1.0 Th. Peptide-spectrum matches (PSMs) were adjusted to a 1% false discovery rate (FDR) with a previously described linear discriminant analysis (83, 84). Filtered PSMs were collapsed to a final protein-level FDR of < 1%. Protein assembly was guided by principles of parsimony to produce the smallest set of proteins necessary to account for all observed peptides (81). For TMT-based reporter ion quantitation, we extracted the summed signal-to-noise (S/N) ratio for each TMT channel and found the closest matching centroid to the expected mass of the TMT reporter ion. MS3 spectra with TMT reporter ion summed signal-to-noise ratios < 100 were excluded from quantitation (85).

### Phosphorylation data processing and analysis

When searching the phosphorylation data, a variable modification for phosphorylation (79.9663 Da) was allowed on serine, threonine, and tyrosine. Searches were performed with a 20-ppm precursor ion tolerance; the product ion tolerance was set to 0.02 Th. Linear discriminant analysis (LDA) was performed to set a PSM FDR of < 1 %. Filtered PSMs were collapsed to a final protein-level FDR of < 1%. Phosphorylation sites were evaluated by an AScore method. Sites with an AScore > 13 were considered localized (86). PSMs with TMT reporter ion summed S/N ratios < 50 were excluded from quantitation.

### Quantification and normalization of TMT data

For each entry (protein or phosphorylation site), the level in each TMT channel was calculated as the log-transformation of the ratio to the median value of all nine TMT channels (three mock, three infected, and three treated) of that entry. Then, every sample was median-normalized so that the log-TMT-ratio was centered at zero. The amounts of phosphorylated proteins were normalized to the total amount of protein by subtracting the log-TMT-ratio of the corresponding protein from the log-TMT-ratio of the phosphorylation site.

### Proteomics differential abundance analysis

Differential abundance analysis of proteins and phosphorylation sites was performed with the Limma v3.42 package in R (87). For protein amounts, log-TMT-ratio was used as input data, and for phosphorylation sites, the site amounts log-TMT-ratio normalized by protein amounts was used. Because the log-TMT-ratio is normally distributed, no voom-normalization was applied. Unequal variance between samples was taken into account with the “arrayWeight” function before fitting the model. *P* values were computed with a moderated t-test, and adjusted *P* values (FDR) were calculated using the Benjamini-Hochberg (BH) correction. Significance for differential expression was determined as an adjusted *P* < 0.1.

### Kinase substrate specificity assays

Reagents used for the peptide library experiments included the Kinase substrate library (Anaspec) and streptavidin-conjugated membranes (Promega). All of the recombinant kinases (SRPK1/2/3, GSK-3α/β, and CK1A/E) were obtained from SignalChem. To determine the substrate motifs, we performed in vitro phosphorylation assays with recombinant kinases on an oriented peptide array library of design Y-A-X_−5_-X_−4_-X_−3_-X_−2_-X_−1_-S_0_/T_0_-X_1_-X_2_-X_3_-X_4_-G-K-K-biotin in the presence of ATP[γ-^32^P]. Reactions were performed in their designated buffers with 20 μM ATP and 0.4 μCi of (33 nM) [γ-^32^P]ATP) at 30°C for 90 min. The peptides were spotted onto streptavidin-coated filter sheets (Promega SAM^2^ biotin capture membrane) and visualized by phosphorimaging on a Typhoon FLA 7000 (33, 34).

### Matrix-processing and substrate-scoring

The matrices were normalized by the sum of the 17 randomized amino acids (all amino acids expect for serine, threonine, and cysteine), to yield a position-specific scoring matrix. The serine, threonine, and cysteine columns were scaled by their median to be 1/17. For scoring substrates, the values of the corresponding amino acids in the corresponding positions were multiplied and scaled by the probability of a random peptide:

ScoreKin X=∏PosPKin XAA, Position1#Random AAlengthpositions

For the percentile score of a substrate by a given kinase, we first computed the a priori score distribution of that kinase by scoring all the reported S/T phosphorylation sites on PhosphoSitePlus (88) (downloaded on January 2020) by the method discussed earlier. The percentile score of a kinase-substrate pair is defined as the percentile ranking of the substrate within the score distribution of the given kinase. This value was used when analyzing all of the detected phosphorylation sites (viral and host) for kinase enrichment.

### Evolutionary conservation analysis

All reference proteomes of the Coronaviridae family of viruses available on Uniprot were downloaded (82 in total, Data file S2). A BLAST search was performed over each proteome using the SARS-CoV-2 N protein as the query; the top hit of each virus was taken to be the N protein. Each designated N protein was then individually aligned to the SARS-CoV-2 N protein with MUSCLE. Using these alignments, the percentage identity was then calculated for each position along the SARS-CoV-2 N protein. All statistical analyses between different conservation of positions were performed with the nonparametric Wilcoxon-Mann-Whitney U test.

### Kinase enrichment analysis

The phosphorylation sites detected in this study were scored by SRPK1/2/3 substrate specificity matrices, and their ranks in the known phosphoproteome score distribution were determined as described earlier (percentile score). For every assessed kinase, phosphorylation sites that ranked within the top 10 percentile within the score distribution were counted as biochemically favored sites for that kinase. Toward assessing SRPK kinase motif enrichment, we compared the percentage of biochemically favored sites in the group of phosphorylated peptides that were decreased in abundance (log_2_Fold Change of −0.75 and below with an FDR of >= 0.1) to the percentage of biochemically favored sites within the set of all detected sites in this study. Statistical significance was determined with Fisher’s exact test.

### Sequence logos

Normalized substrate specificity matrices were scaled to represent relative probability (each position sum up to 1), and probability sequence logos were generated with the ggseqlogo v0.1 (89) package in R.

### Plasmids

The ACE2-pLEX overexpression plasmid was generated by subcloning a synthesized gBlock of the ACE2 ORF (Ref. seq. NM_001371415.1) into the pLEX plasmid with HiFi DNA Assembly (NEB Cat# M5520AA) and subsequent bacterial transformation. Bacterial colonies were then selected and plasmid DNA isolated with the Genejet Plasmid Miniprep kit (Thermo Fisher Cat# K0503). Plasmids were then sequenced by Sanger sequencing to confirm successful cloning of the ACE2 ORF. Lentiviruses were packaged as per standard protocols with a VSV-G envelope protein, and A549 or A549-Cas9 cells were transduced and selected with puromycin (2 μg/ml).

### Treatment of cells with siRNA

ACE2-A549 cells were treated with 30 μM siRNA (SRPK1-specific: Horizon M-003982-02-0010; Nontargeting: Thermo 4390843 or Horizon D-001206-13-05) according to the HiPerfect (Qiagen) Fast-Forward protocol and plated in 6-well plates. After 2 days, cells were trypsinized and replated in 24-well plates and re-transfected with 50 μM siRNA using the same protocol. After 2 additional days, the cells were infected with SARS-CoV-2 at an MOI of 0.005 for 1 hour in 250 μl of DMEM, 2% FBS for testing viral RNA. After 1 hour of infection, 250 μl of DMEM, 2% FBS was added to the inoculum and cells were incubated for 24 hours. The medium was then removed, and the cells were lysed with 500 μl of TriZol. To prepare cell lysates for analysis on Phos-tag gel, cells that were transfected with siRNA as described earlier were infected with SARS-CoV-2 at an MOI of 0.5 for 1 hour. Then, 250 μl of DMEM, 2% FBS was added to the inoculum and the cells were incubated for 24 hours. The medium was then removed, and the cells were washed once with 0.5 ml of PBS, lysed with 200 μl of 1x Laemmli buffer, boiled for 15 min, and removed from the BSL3.

### Quantitative reverse-transcription PCR

Cells were resuspended in TRIzol Reagent (Thermo Fisher Cat #15596018) and total RNA was isolated by either phase separation with chloroform and isopropanol or with the Zymo Direct-zol RNA Miniprep kit (Zymo Cat #R2050). One-step qRT-PCR was performed with the Invitrogen EXPRESS One-Step Superscript qRT-PCR kit (Thermo Fisher Cat #11781200) and the commercial Taqman probe ACE2 (Hs01085333_m1). The SARS-CoV2 primer-probe set was synthesized with IDT DNA based on the sequences provided by the CDC “Research Use Only 2019-Novel Coronavirus (2019-nCov) Real-time RT-PCR Primers and Probes” set N1. For qRT-PCR analysis of *SRPK1* expression, a commercial Taqman probe (Hs00177298_m1) was used. For infected 229E cells, RNA was extracted with an NEB Monarch total RNA miniprep kit (T2010S). To quantify viral RNA, we used a commercial Taqman probe CoV_229E (Vi06439671_s1). Reactions were cycled on the Applied Biosystems QuantStudio 3 Real-Time PCR System and analyzed with QuantStudio software version v1.4.1. RNA abundance was normalized to that of endogenous 18S, which was analyzed with a specific primer-probe set (Thermo Fisher Cat #4319413E).

### Western blotting

To determine the extent of SRPK1 knockdown, cells were trypsinized, removed from plates, and pelleted by centrifugation at 3000*g* for 4 min. Cell pellets were washed once with PBS. Cells were lysed by resuspension in RIPA buffer [10 mM Tris-HCl (pH 7.5), 1 mM EDTA (pH 8.0), 1% Triton X-100, 0.1% sodium deoxycholate, 140 mM NaCl, 0.1% SDS] and incubation at 4°C for 15 min. Chromosomal DNA was sheared by passing the lysates through an insulin syringe, and cellular debris was removed by centrifugation at 21,100 *g* for 10 min at 4°C. The total protein concentration of the cellular lysates was determined by Bradford assay, and all samples were normalized to a concentration of 1 µg/µl. Cellular lysates were separated by SDS-PAGE using 15 to 20 µg of total cellular protein per lane [4 to 20% Mini-PROTEAN TGX gels (BioRad), 120 V for 1 hour]. Proteins were subsequently transferred to nitrocellulose membranes (90 V for 1 hour at 4°C). Nitrocellulose membranes were blocked for at least 3 hours with PBST containing 5% milk. For detection of SRPK1, membranes were incubated with primary antibody (BD cat. No. 611072, 0.025 µg/ml) in PBST containing 5% milk overnight at 4°C. For detection of GAPDH, membranes were incubated with primary antibody (Cell Signaling, cat. No. 2118S, 1:10,000 dilution) in PBST containing 5% milk overnight at 4°C. Anti-mouse (Invitrogen cat. No. A16072) and anti-rabbit (Thermo Scientific cat. No. A16104) secondary antibodies were used at 1:20,000 and 1:10,000 dilutions, respectively, in PBST containing 5% milk for 1 hour at room temperature. Blots were developed with Clarity Max ECL substrate (BioRad). To determine the amounts of phosphorylated and unphosphorylated N protein, Western blotting analysis of lysates resolved on Phos-tag gels were used. 15 μl of ladder or sample with MgCl_2_ added to a final concentration of 1 mM was loaded into a SuperSep Phos-tag gel (Wako 192-18001). Gels were run at 30 mA, which was followed by four 20-min washes in transfer buffer with 10 mM EDTA. Gels were then washed for 10 min in transfer buffer without EDTA and transferred at 60V for 60 min. Blots were blocked for 1 hour in PBST, 5% milk at room temperature and then incubated with anti-N antibody (Genetex- GTX635679, 1:5,000 dilution) in PBST, 5% milk overnight at 4°C. Anti-rabbit secondary antibody was used at a 1:10,000 dilution, and blots were developed with ECL substrate (Bio-Rad). As a control, Western Blots were also performed with lysate loaded into a SDS PAGE gel. Samples were loaded as described earlier, then transferred without EDTA wash at 60V for 60 min. The blotting procedure used was identical to that for the Phos-Tag gel. For Western blotting analysis of GAPDH, blots were stripped with glycine, 0.1% SDS, 1% Tween 20 (pH 2.2). Blocking and antibody incubation then proceeded as described earlier. Western blot images were quantified with ImageJ software. Each band on a given phos-tag N protein Western blot was quantified three independent times. We calculated the percentage phosphorylated and unphosphorylated protein based on these measurements and then averaged the three technical replicates together to determine the relative percentage of phosphorylated to unphosphorylated N protein for each biological replicate. We repeated this process for all three biological replicates.

### Crystal violet staining

To visualize the cytopathic effect of A549 cells expressing ACE2 or not, cells were infected with SARS-CoV-2 at an MOI of 0.1 for 1 hour at 37℃, after which, 0.5 ml DMEM, 2% FBS was added to each well and the infection was allowed to continue for 72 hours. The monolayer of cells was then stained with 0.1% Crystal violet in 10% NBF for 15 min. The stain was then removed, and the cells were rinsed with ultra-pure water and then imaged.

### SRPK inhibitor treatment assays

Three different inhibitors of SRPK1 and SRPK2 were used to assay effects on viral replication: SRPIN340, SPHINX31, and Alectinib (MedChem Express). SRPIN340 and SPHINX were suspended in DMSO at 1000X the final concentration required, whereas Alectinib was resuspended at 500X. The inhibitors were then diluted to the appropriate concentrations in cell-specific medium. Cells were treated with inhibitor 12 hours before they were infected. Cells were then infected for 1 hour as described earlier. After infection, medium with 2% FBS and either drug or DMSO as a control was added to each well (for qRT-PCR and Calu-3 microscopy assays) or the inoculum was removed and medium with 2% FBS and either drug or DMSO was added to each well (for infectious titer quantification).

### Cytotoxicity assays

A549-Cas9 ACE2, Calu-3, and Huh7 cells were treated with the concentrations of SRPIN-340, SPHINX-31, or Alectinib indicated in the figure legends in a consistent volume of vehicle (DMSO). Forty-eight hours later, samples were processed according to the CellTiter-Glo (Promega) protocol and luminescence was determined with a luminometer.

### Calu-3 cell infections and immunofluorescence assays

To infect Calu-3 cells for immunofluorescence assays, the cells were treated with DMSO or 53 μM SRPIN340 for 12 hours before infection. To infect Calu-3 cells, the medium was removed, and the cells were washed with 0.5 ml of PBS. Virus was added at an MOI of 2.5 and the cells were infected at 37°C for 1 hour. After infection, 200 μl of medium and 2x SRPIN340 or DMSO was added to cells and incubated at 37°C for 24 hours. To fix the cells, the culture plate was submerged in 10% NBF for 2 hours. The NBF was removed, and the cells were placed in PBS. For immunostaining, cells were permeabilized with 0.1% Triton X-100 and blocked in PBS, 5% BSA, and 0.1% Tween-20. Cells were stained with either an anti-dsRNA antibody (J2, Sigma MABE1134) at a 1:125 dilution or an anti-SARS-CoV-2 Spike RBD protein antibody (ProSci #9087) at a 1:150 dilution. Alexa Fluor 488– or Alex Fluor 594–conjugated secondary antibodies were used at a 1:1000 dilution. DNA was stained with Hoechst 33342 dye at a 1:10,000 dilution in PBS. Images were captured with a Bio-Rad Zoe fluorescent cell imager.

### In vitro SRPK1/2 phosphorylation assays

Recombinant N protein (2 μM) was incubated with the recombinant kinases SRPK1 (80 nM), GSK-3A (5 nM), or CK1ε (30 nM) in Kinase Buffer 1 (SignalChem) for 15 min at 30°C. Reactions were terminated by the addition of SDS loading buffer. The reaction mixtures were resolved by phos-tag gel and analyzed by Western blotting with monoclonal anti-N protein antibody (GeneTex) as described earlier. For autoradiography, the reactions were supplemented with γ[^32^P]-ATP and were resolved by SDS-PAGE. The N protein bands (48 kDa) were excised, and radioactivity was measured by Typhoon FLA 7000.

### Human lung dissociation, and purification, culture, and immunostaining of primary type two pneumocytes

Human lung tissues from 6 different donors (2 females and 4 males aged 20 to 49) were washed with PBS containing 1% Antibiotic-Antimycotic and cut into small pieces. Samples were then digested with enzyme mixture (Collagenase type I: 1.68 mg/ml, Dispase: 5 U/ml, DNase: 10 U/ml) at 37°C for 1 hour with rotation. The cells were filtered through a 100-µm strainer, rinsed with 15 ml of DMEM/F12, 10% FBS, and centrifuged at 450*g* for 10 min. The supernatant was removed, and the cell pellet was resuspended in red blood cell lysis buffer for 10 min, washed with DMEM/F12, 10% FBS, and filtered through a 40-µm strainer. Total cells were centrifuged at 450*g* for 5 min at 4ºC and the cell pellet was processed for AT2 purification. To purify human type two pneumocytes, approximately 2 to 10 × 10^6^ total human lung cells were resuspended in MACS buffer and blocked with Human TruStain FcX (Biolegend cat# 422032) for 15 min at 4ºC, which was followed by staining with HTII-280 antibody (Terrace Biotech, TB-27AHT2-280) for 1 hour at 4ºC. The cells were washed twice with MACS buffer, which was followed by incubation with anti-mouse IgM microbeads for 15 min at 4ºC. The cells were loaded into the LS column and collected magnetically. AT2 cells (3 x 10^3^) were resuspended in serum-free medium, mixed with an equal amount of Matrigel (Corning Cat# 354230), and plated as drops on 6-well plates. The medium was changed every three days. Alveolospheres were passaged every 14 days. For virus infection experiments, human AT2 cells were seeded at 10 x 10^4^ cells per insert on 5% Matrigel–coated Transwells with 0.4-μm pore-sized inserts (Corning). Cells were treated with DMSO or 53 μM SRPIN340 12 hours before infection with medium at the top and bottom of the transwell. To infect the cells, the medium was removed from the top of the transwell, and the cells were washed with 200 μl of PBS. The cells were then infected at an MOI of 1 for 1 hour. Virus was then removed, and the cells were cultured for 24 hours. To fix cells and remove them from the BSL3 facility, plates and wells were submerged in 10% NBF for 2 hours. The NBF was removed, and the cells were placed in PBS until needed for staining. For immunostaining of cell cultures, the membrane from the transwell insert was removed with a scalpel and washed with PBS, permeabilized in PBST (0.1% Triton X-100 in PBS), and incubated with blocking buffer (1% BSA in PBST) for 1 hour at room temperature. Samples were incubated with primary antibodies: Prosurfactant protein C (1:500, Millipore, cat# AB3786) and SARS-CoV-2 spike protein (1:500, Genetex cat# GTX632604) in blocking buffer at 4°C overnight. Membranes were then washed three times in PBST, incubated with secondary antibodies in blocking buffer for 1 hour at room temperature, washed three times with PBST, and mounted with Fluor G reagent with DAPI. All confocal images were collected with an Olympus Confocal Microscope FV3000 using a 40X objective lens.

### Statistical analysis

For evolutionary conservation analysis, a nonparametric Wilcoxon-Mann-Whitney U test was used on the percentage identity values of the corresponding amino acids (that is, the percentage of coronaviruses for which that amino acid is conserved). For differential expression analysis of proteins and phosphorylation sites, *P* values were computed using a moderated *t* test (limma-based), and adjusted *P* values (FDR) were calculated using the Benjamini–Hochberg (BH) correction. For all biological experiments, nonparametric Wilcoxon-Mann-Whitney U test was used unless specifically indicated otherwise.

## Supplementary Material

Supplementary Material

SM Excel file 1

SM Excel file 2

SM Excel file 3

SM Excel file 4

SM Excel file 5

Reproducibility checklist
