# Supplementary material for: Host protein kinases required for SARS-CoV-2 nucleocapsid phosphorylation and viral replication
Source: Sci Signal. Author manuscript; Available in PMC 2023 Jan 10. (PMC9830954; doi:10.1126/scisignal.abm0808)
Supplement: Supplementary Material [file NIHMS1851690-supplement-Supplementary_Material.docx]

**­­
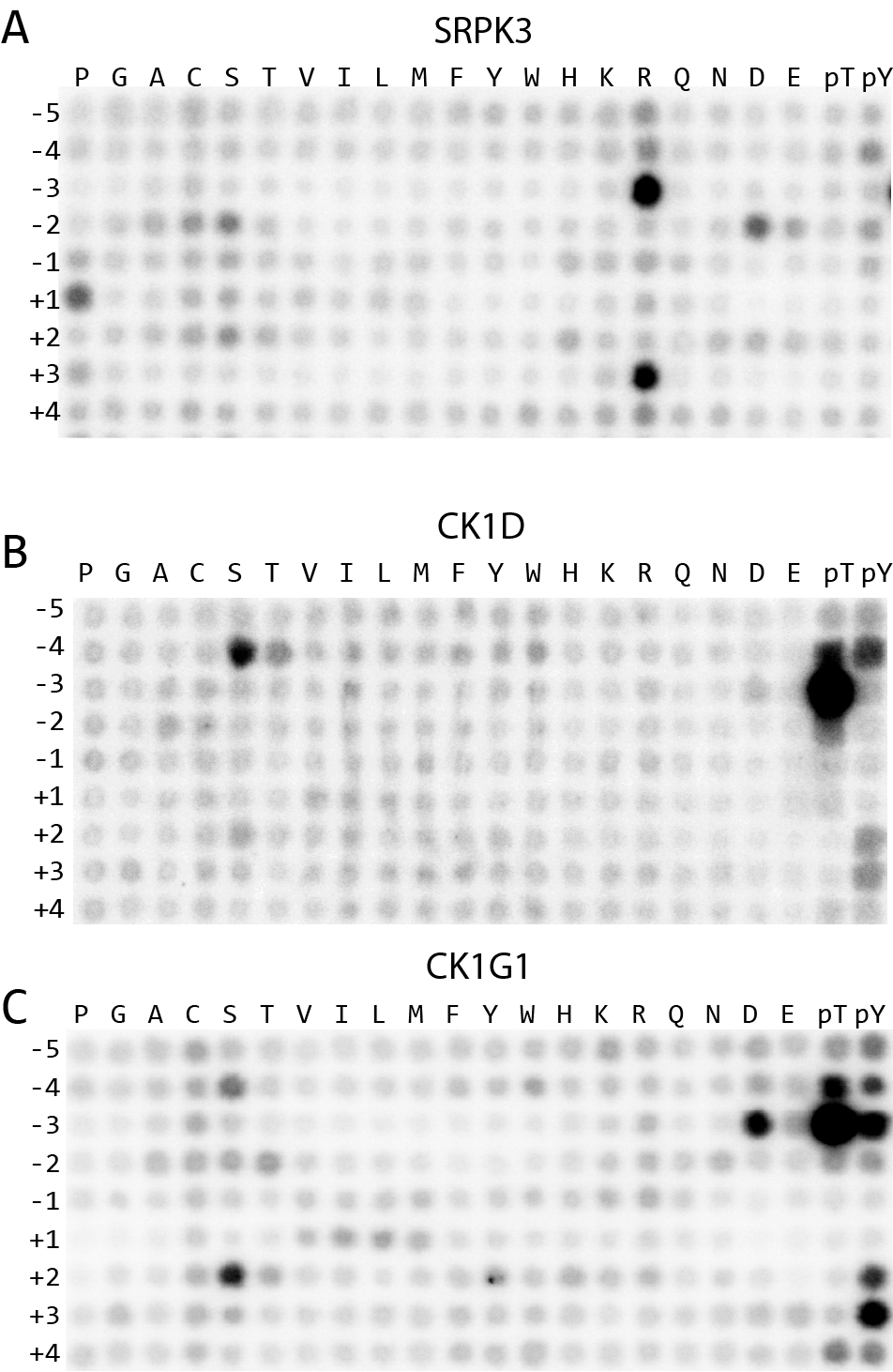
**

**Fig. S1. Biochemical substrate specificities of SRPK3, CK1D, and CK1G1.** The biochemical specificity of SRPK3, CK1D, and CK1G1 was determined with combinatorial peptide libraries (images are representative of at least two replicate experiments). (**A**) Similarly to SRPK1/2, SRPK3 was also selective for arginine residues at the -3 and +3 positions, serines at the -2 and +2 positions, and proline at the +1 position. (**B** and **C**) Similarly to CK1A/ε, CK1D and CK1G1 were also selective for phosphoserine and phosphothreonine residues at position -3 and for serine at position -4.

**Fig. S2. Sequence logos of the SRPK, GSK3, and CK1 families based on their biochemical substrate specificity matrices.** The values of the normalized substrate specificity matrices shown in Fig. 1 and Fig. S1 were converted into relative probabilities and plotted as a sequence logo. For the GSK3 and CK1 families, phosphorylated residues are also included.


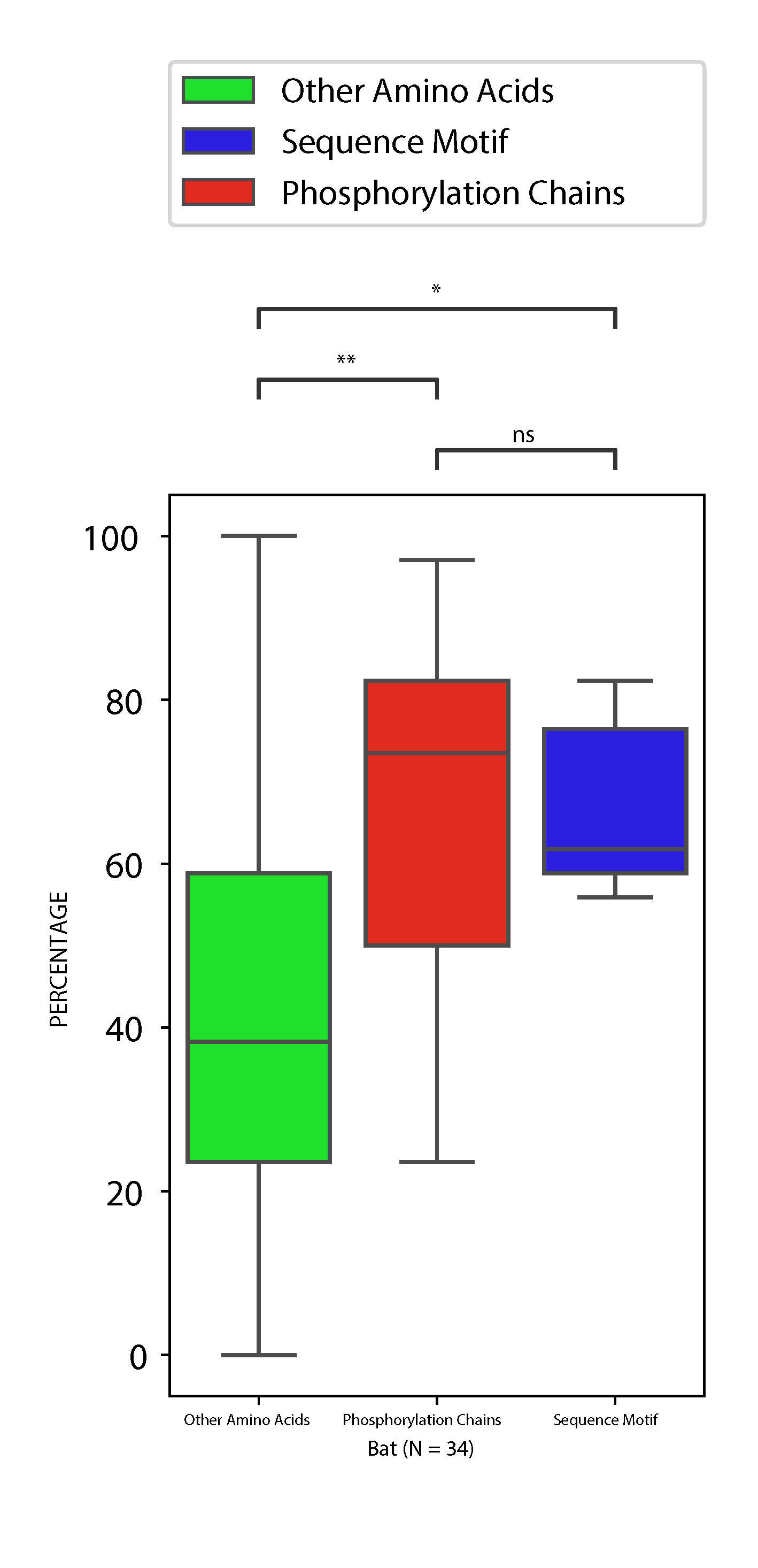


**Fig. S3. Evolutionary conservation across 34 bat coronaviruses.** Evolutionary conservation comparison of three types of amino acid residues in the SR-rich domain of the N protein across 34 bats coronaviruses. Sequence motif: amino acid residues predicted to be essential for the substrate specificity of the priming sites (Ser^188^: Arg^185^/Ser^186^/Ser^190^/Arg^191^; Ser^206^: Arg^203^/Pro^207^/Arg^209^); phosphorylation chains: phosphorylation sites described in the phosphorylation model in Fig. 2C; other amino acids: all other amino acid residues in the SR-rich domain. Mann-Whitney U test: **P* < 0.05, ***P* < 0.01; ns, not significant.

**
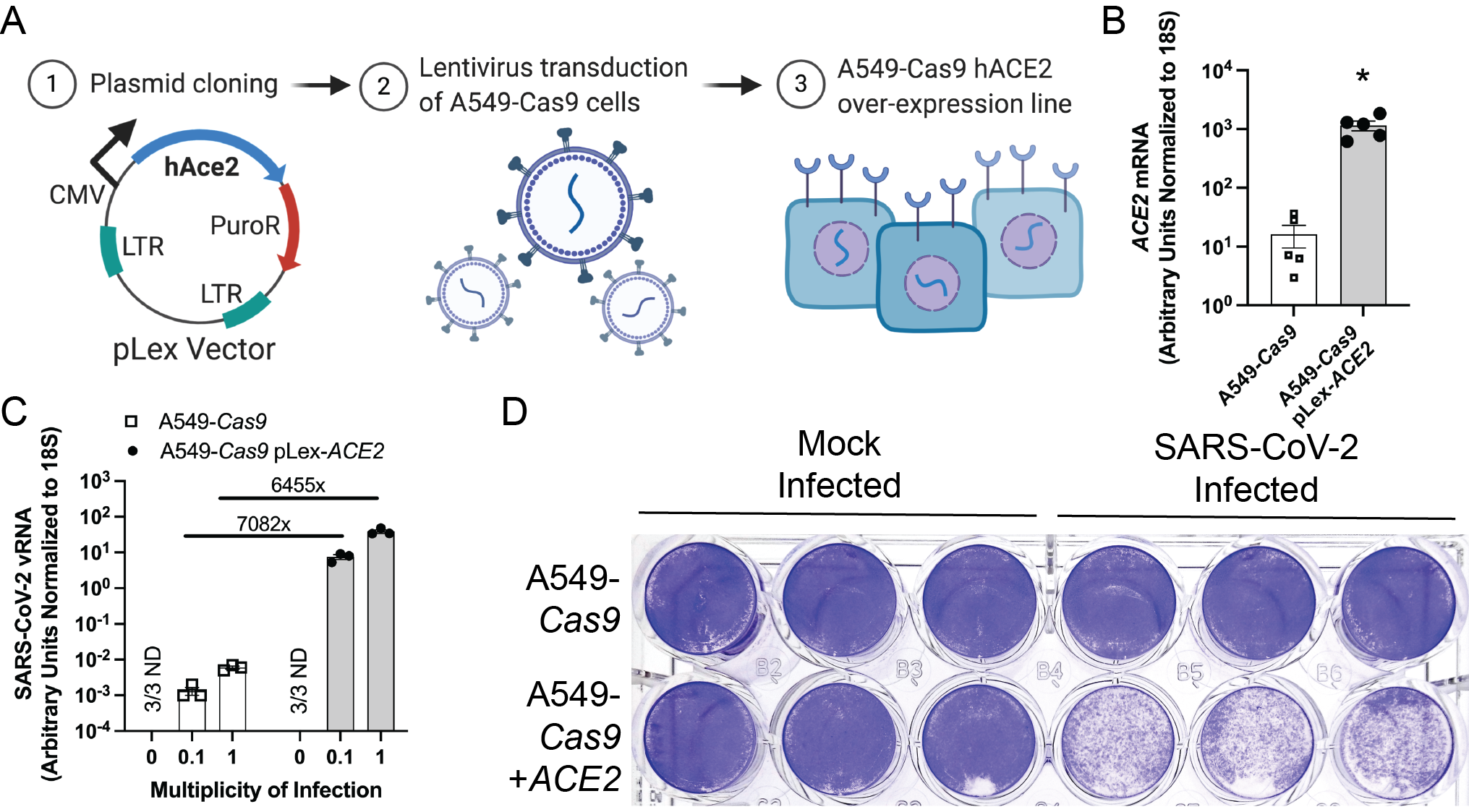
**

**Fig. S4. Development and validation of an A549-ACE2 line for SARS-CoV-2 infection.** (**A**) Scheme for generation of the A549-ACE2 cell line. (**B**) Quantification of *Ace2* mRNA abundance by qRT-PCR analysis of the parental A549 cells and the A549 cells transduced with the pLex-ACE2 lentivirus. Data are means ± SEM of five independent biological replicates. **P* < 0.05. (**C**) Quantification of SARS-CoV-2 RNA abundance in A549 and A549-*ACE2* cells 24 hours after infection with virus at the indicated MOIs. Data are means ± SEM of three independent biological replicates. (**D**) Crystal violet staining of A549 and A549-ACE2 cells 72 hours after infection with SARS-CoV-2 at an MOI of 0.1. Data are representative of two experiments. Data were analyzed by nonparametric Wilcoxon Mann-Whitney U exact test, unless otherwise stated, and measurements were taken from distinct samples.

**Fig. S5. Proteomics analysis of Alectinib-pretreated, infected ACE2-A549 cells.** Protein amounts in ACE2‑A549 cells, comparing infected cells with Alectinib pretreatment to infected cells with no pretreatment (n = 3 experiments). Adjusted *P* values (FDR) were computed by moderated *t* test and adjusted with Benjamini-Hochberg correction: *FDR < 0.1, **FDR < 0.05, ***FDR < 0.01; ns, not significant.


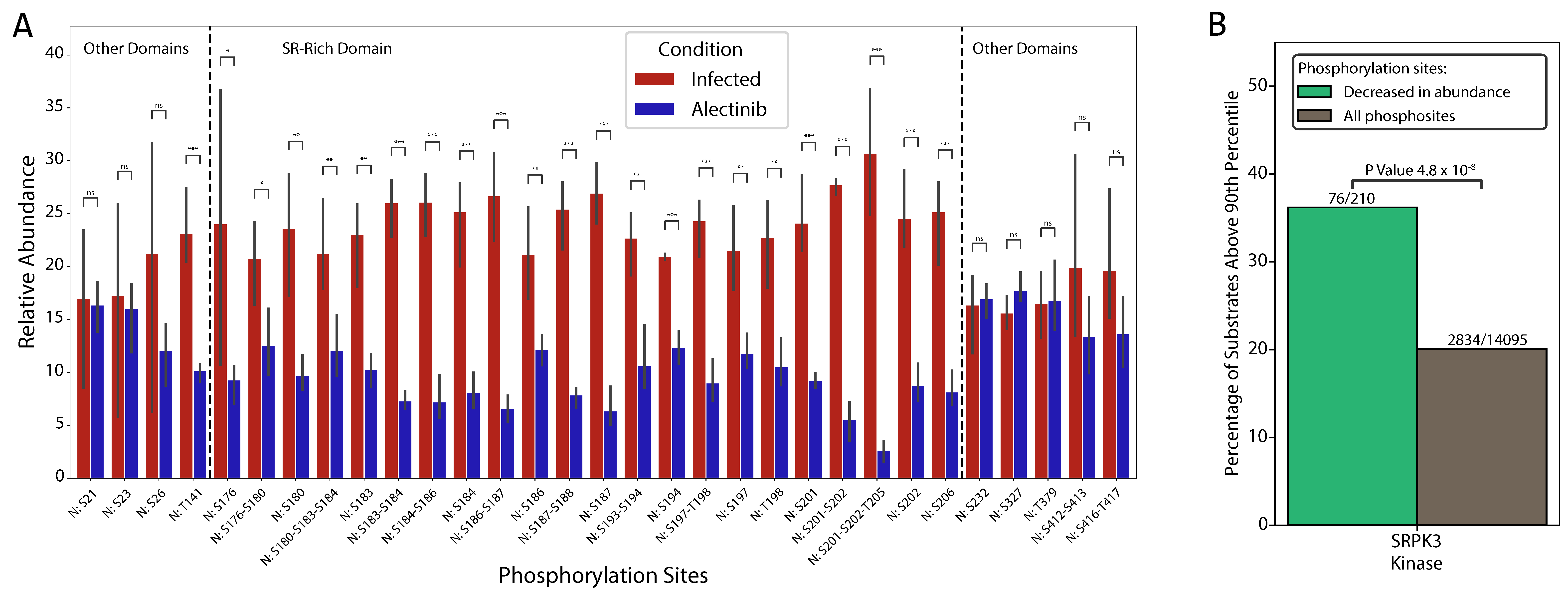


**Fig. S6. Phosphoproteomic analysis of Alectinib-pretreated, infected Vero E6 cells.** (**A**) Phosphorylation site abundances (normalized by protein amount) in Vero cells upon Alectinib treatment (n = 3 experiments). Adjusted *P* values (FDR) were computed by moderated *t* test and adjusted with Benjamini-Hochberg correction: *FDR < 0.1, **FDR < 0.05, ***FDR < 0.01. (**B**) SRPK3 enrichment analysis for the phosphorylation sites that were decreased in abundance upon Alectinib treatment in A549-ACE2 cells. Denoted *P* values were computed with Fisher’s exact test.
